# Strength of activation and temporal dynamics of bioluminescent-optogenetics in response to systemic injections of the luciferin

**DOI:** 10.1016/j.neuroimage.2024.120882

**Published:** 2024-10-02

**Authors:** Emily F. Murphy, Aniya Means, Chen Li, Hector Baez, Manuel Gomez-Ramirez

**Affiliations:** a Department of Brain and Cognitive Sciences, University of Rochester, Rochester, NY 14642, USA; b The Ernest J. Del Monte Institute for Neuroscience, University of Rochester School of Medicine and Dentistry, Rochester, NY 14642, USA; c Center for Visual Science, University of Rochester, Rochester NY 14642, USA

**Keywords:** Optogenetics, Bioluminescence, Mouse, Fluorescence, Luciferin, Luciferase

## Abstract

BioLuminescent OptoGenetics (“BL-OG”) is a chemogenetic method that can evoke optogenetic reactions in the brain non-invasively. In BL-OG, an enzyme that catalyzes a light producing reaction (i.e., a luciferase) is tethered to an optogenetic element that is activated in response to bioluminescent light. Bioluminescence is generated by injecting a chemical substrate (*luciferin*, e.g., h-Coelenterazine; h-CTZ) that is catalyzed by the luciferase. By directly injecting the luciferin into the brain, we show that bioluminescent light is proportional to spiking activity, and this relationship scales as a function of luciferin dosage. Here, we build on these previous observations by characterizing the temporal dynamics and dose response curves of bioluminescence generated by luminopsins (LMOs), a proxy of BL-OG effects, to intravenous (IV) injections of the luciferin. We imaged bioluminescence through a thinned skull of mice running on a wheel, while delivering h-CTZ via the tail vein with different dosage concentrations and injection rates. The data reveal a systematic relationship between strength of bioluminescence and h-CTZ dosage, with higher concentration generating stronger bioluminescence. We also found that bioluminescent activity occurs rapidly (< 60 s after IV injection) regardless of concentration dosage. However, as expected, the onset time of bioluminescence is delayed as the injection rate decreases. Notably, the strength and time decay of bioluminescence is invariant to the injection rate of h-CTZ. Taken together, these data show that BL-OG effects are highly consistent across injection parameters of h-CTZ, highlighting the reliability of BL-OG as a minimally invasive neuromodulation method.

## 1. Introduction

Methods of neuromodulation can provide fundamental insight into the role that neural ensembles play in mediating perceptual functions and motor actions. In a seminal study, Salzman and colleagues (Salzman et al., 1990) solidified the role of medio-temporal (MT) cortex in driving perception of visual motion by showing that intracortical microstimulation (ICMS) in MT systematically biases visual motion judgements. ICMS was also used to show that perception of flutter (i.e., low frequency vibrations) is mediated, at least in part, by neurons in primary somatosensory cortex (SI) (Romo et al., 1998). ICMS has become one of the leading methods used in brain computer interface (BCI) applications, especially in clinical applications for human patients that rely on neuroprosthetic devices (Ajiboye et al., 2017; Chaudhary et al., 2022; Collinger et al., 2014, 2013; Downey et al., 2016). Yet, although ICMS has led to fundamental discoveries, the technique can only provide uni-directional neuromodulation effects by upregulating activity of neural populations (i.e., it cannot be used to cause inhibitory effects). Further, ICMS can generate non-specific effects by modulating widespread networks through stimulation of passing fibers (Histed et al., 2009; Kumaravelu et al., 2022). ICMS also fails to provide selective modulation of cell-type specific populations (e.g., inhibitory vs. excitatory cells). Indeed, understanding how individual cell types contribute to a particular neural or perceptual function can lead to fundamental insight into the circuit mechanisms that mediate such function.

Neuromodulation methods that rely on genetic modification strategies (e.g., optogenetics and chemogenetics) can provide precise excitatory and/or inhibitory modulation of cell-type specific circuits (Aston-Jones and Deisseroth, 2013; Bernstein et al., 2012; Bernstein and Boyden, 2011). In particular, optogenetics affords high spatio-temporal precision, even at the single cell level (Marshel et al., 2019). However, similar to ICMS, optogenetics requires an invasive surgical procedure to generate neuromodulation effects in the brain (e.g. a cranial surgery to install a chronic implant to support the external light source and provide optical access to the brain). In contrast, chemogenetic methods are minimally invasive, largely because they do not require a cranial implant to generate neuromodulation (Berglund et al., 2016; Dimidschstein et al., 2016; Gomez-Ramirez et al., 2020; Hori et al., 2023; Song et al., 2022; Urban and Roth, 2015; Vlasov et al., 2018; Zhang et al., 2022). Further, chemogenetics can modulate activity across broad spatial scales by systemically injecting the chemical that drives the chemogenetic molecule, a useful feature for large-brain animals such as non-human primates (Cushnie et al., 2023; Hori et al., 2023; Raper and Galvan, 2022). However, chemogenetics has less temporal precision, in comparison to optogenetics and ICMS, with some methods producing effects that last hours as in the case of designer receptors exclusively activated by designer drugs (DREADDs) (Alexander et al., 2009; Claes et al., 2022). Thus, all neuromodulation methods come with inherent advantages and disadvantages, and it is incumbent on the researcher to use the approach (or approaches) best suited to answer the question of the study.

Our recent work (Gomez-Ramirez et al., 2020) and others (Berglund et al., 2020, 2016; Crespo et al., 2021; Petersen et al., 2022; Prakash et al., 2022; Tung et al., 2015; Zhang et al., 2020) demonstrated a hybrid optogenetic and chemogenetic method that produces optogenetic modulation using internally-generated bioluminescence. This method, termed BioLuminescent-OptoGenetics (BL-OG), tethers an enzyme (i.e., luciferase) to an opsin (a construct known as luminopsin; LMO) that is activated by bioluminescence when a luciferase catalyzes a luciferin. New generations of LMOs (e.g., LMO7) operate on Förster resonance energy transfer (FRET) by tethering the luciferase to a fluorescent protein to generate enhanced photonic activity to better drive the opsins. Indeed, a recent study showed lower bioluminescence but stronger BL-OG effects on spiking activity in cells that use FRET to drive opsins (i. e., LMO7 vs. LMO3) (Björefeldt et al., 2024). Study also show higher photonic activity in cells that express a light emitter without an opsin vs. cells that express an LMO (e.g., LMO3 or LMO7), which highlights the coupling efficiency between light-emitting molecule and the optogenetic element. We previously showed that the bioluminescence and BL-OG effects of the LMO3 molecule is highly proportional to injections of the substrate (Gomez-Ramirez et al., 2020). Here, we study whether the bioluminescence response of the LMO7 molecule is also commensurate to the injection parameters of its substrate, h-CTZ.

The rapid catalytic reaction that produces bioluminescence can generate relatively short-term effects (e.g., minutes to tens of minutes). BL-OG can also provide moment-to-moment neuromodulation by conventional activation of opsins using a fiber optic cable. A critical feature of BL-OG is that the bioluminescent light created from the BL-OG mechanism provides an optical readout of the neuromodulation effect itself (Gomez-Ramirez et al., 2020). We recently showed that bioluminescent light is directly proportional to multi-unit spiking activity, and this relationship scales as a function of CTZ amount (Gomez-Ramirez et al., 2020). Taken together, BL-OG represents a highly feasible and handy method for generating and tracking neuromodulation effects in cell-type specific circuits via minimally invasive approaches.

To further establish and facilitate the use of BL-OG as a minimally invasive modulation method, it is key to establish the temporal properties and strength of the neuromodulation effects of BL-OG in response to different injection parameters of the luciferin. Here, using bioluminescence as a proxy for spiking activity (Gomez-Ramirez et al., 2020), we quantified the temporal dynamics and dose response functions of the LMO7 molecule to different concentrations and rate of injections of the luciferin, h-Coelenterazine (h-CTZ). Activity was imaged through the thinned-skull of a mouse to further highlight the minimally invasive properties of BL-OG. We found that bioluminescence increases linearly as a function of h-CTZ dosage, and, importantly, the overall strength of bioluminescence is consistent across the rate of injection. We also observed that the onset of bioluminescence in neocortex occurs rapidly (~10 s after h-CTZ injection), regardless of h-CTZ concentration. However, as expected, the onset time of bioluminescence is systematically delayed as a function of injection rate. Taken together, our data show that bioluminescence response, and, by extension, BL-OG effects (Gomez-Ramirez et al., 2020), are highly predictable across injection parameters of the luciferin, highlighting the consistency and feasibility of BL-OG as a chemogenetic neuromodulation method.

## 2. Methods

### 2.1. Animals

Experiments were conducted using C57/BL6 mice (*N* = 17 across all experiments; The Jackson Laboratory stock #000,664) bred in the vivarium at the University of Rochester. All experimental methods are consistent with National Institutes of Health guidelines and approved by the Institutional Animal Care and Use Committee at the University of Rochester (UCAR).

### 2.2. Molecular construct and surgical procedures

**Fig. 1.**
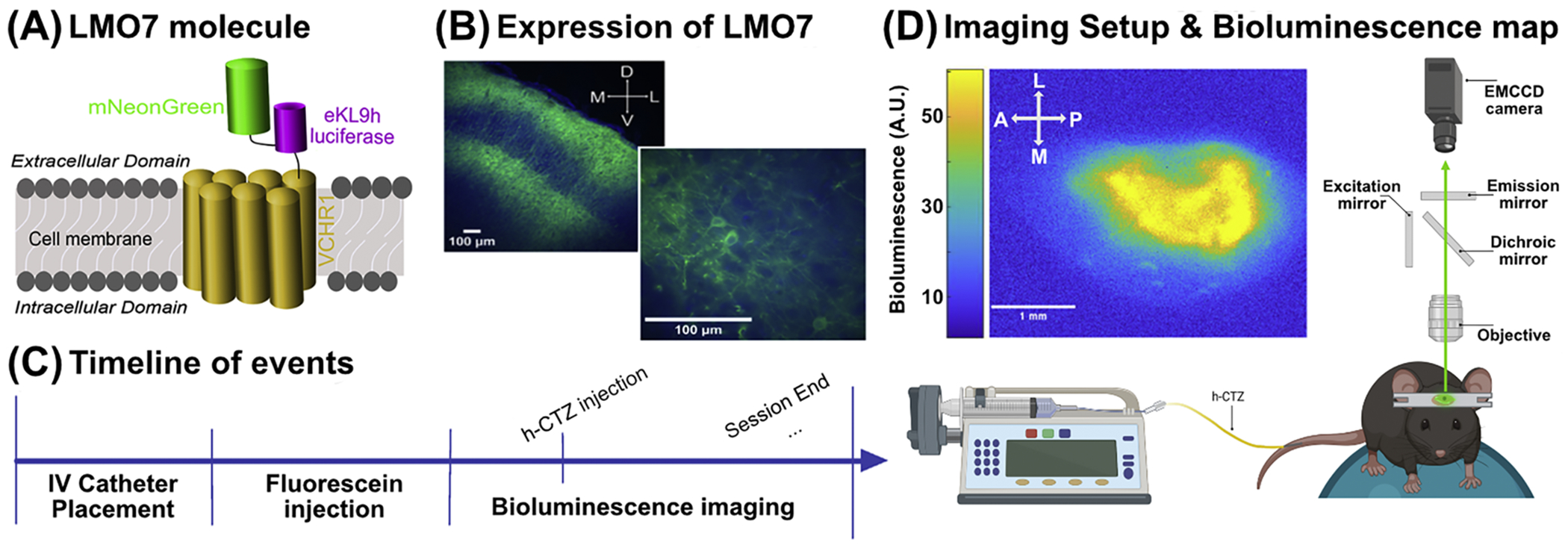
**(A)** Schematic illustration of the LMO7 molecule. The LM07 molecule comprises a fused fluorescent and bioluminescent protein, known as NCS2 (mNeonGreen-eKL9h, a modified Oplophorus-based luciferase), that is tethered to the N-terminus of Volvox Channelrhodopsin 1 (VChR1). The LMO7 molecule is anchored to the channelrhodopsin embedded in the cell membrane with the NCS2 molecule residing in extracellular space. **(B)** Representative histological images showing fluorescence expression in the left SI of a mouse. The lower panel shows a zoomed image highlighting expression of LM07 around the cell membrane. The blue oval-like shapes represent DAPI. **(C)** Illustration of the timeline of events within a single day of imaging. The session began with placing a catheter in the tail vein of the mouse, and then injecting fluorescein to verify that the catheter has access to the IV space. After fluorescein washed out (i.e., fluorescence was not present), bioluminescence commenced. After 10 min of bioluminescence imaging, we injected h-CTZ via the catheter and imaged for another 50 min. We continue running imaging sessions until all concentrations of h-CTZ were injected for that current day. **(D)** Schematic of the experimental setup used to image bioluminescence in the mouse. The image on the left side shows a representative example of a bioluminescence color map in response to a 3.93 mM concentration of h-CTZ via the tail vein.

The luminopsin-7 (LMO7) molecule comprises a fused fluorescent and bioluminescent protein, known as NCS2 (mNeonGreen-eKL9h, a modified Oplophorus-based luciferase), that is tethered to the N-terminus of Volvox Channelrhodopsin 1 (VChR1; **see** Fig. 1A) (Björefeldt et al., 2024). LMO7 was encoded within an adeno-associated viral 1 (AAV-1) construct under the human synapsin (h-Syn) promoter (titer >10^13^ gc/ml). The virus was purchased from VectorBuilder (Catalog # P200603–1009amb).

All surgical procedures were conducted under general and local anesthesia. Mice were anesthetized with isoflurane (3 % for induction and 1–2 % for the remainder of the surgical procedure) and stereotaxically fixed. The fur was removed from the top of the head. The area was treated with 4 or 5 % lidocaine cream, and then aseptically prepared. A custom-made head post was affixed to the skull using C&B Metabond^®^ Quick Adhesive Cement (Parkell). The skull was shaved down until the cranium was translucent throughout a 3 mm radius in the area of left primary somatosensory cortex (SI; −1.25 AP and +3.5 mm ML from the Bregma). Skull thinning was performed using a modified approach established in mice (Steinzeig et al., 2017). A NeoBurr sterile carbide bur drill bit (Microcopy, FG-4) was used to shave the skull until the cranium was translucent. The drill bit was lightly brushed across the skull in a circular and side-to-side motion every two seconds to achieve a smooth surface and avoid overheating of the skull. The skull was regularly flushed with cooled saline to increase visibility and reduce heating from the drilling. The virus with the LMO7 was injected through three burr holes in the thinned skull, 400 nl in each burr hole at an injection rate of 10 nl/minute and a depth of 400 μm through custom-pulled micropipettes using a Stoelting^™^ Quintessential Stereotaxic Injector (QSI). The thinned skull and burr holes were then covered with clear nail polish (Electron Microscopy Sciences, Manufacturer Part Number: 72,180). Mice were imaged at a minimum of two weeks after the surgery to allow time for the virus to express in the brain (Fig. 1B).

### 2.3. Luciferin injections

Bioluminescence was produced using the substrate h-Coelenterazine-SOL (h-CTZ) for in vivo applications (NanoLight Technology, CAT#3011). Vials were stored at −80 °C until shortly before use. Once removed from the −80 °C freezer, the h-CTZ vial was wrapped in aluminum foil to protect it from light, and left on the lab counter for a few minutes to reach room temperature. One hundred microliters of sterile water was added to each vial, with vials placed in a 55 °C water bath for the powder to dissolve (Crespo et al., 2021). An additional two hundred microliters of sterile saline for injection was then added to the vial to form a stock solution of 300 μL volume. For the Dose Response experiment, different levels of h-CTZ concentration (of the same volume) were derived by further diluting the stock solution with different amounts of sterile saline (e.g., 10 μL and 244 μL of sterile saline to achieve 3.93 mM and 0.1 mM dosage concentrations of 250 μL, respectively). For each concentration condition, 250 μL of prepared solution was pre-loaded into a microbore tubing extension set (Smith’s Medical, 536040C), with one end connected directly to the tail vein catheter and the other end connected to another extension tubing filled with sterile water for injection. The end tube was connected to an infusion pump (Harvard Apparatus, Model 11) that controlled the rate of injection of the luciferin. For the Dose Response experiment, we injected h-CTZ with six different concentrations (0.74, 0.98, 1.72, 2.46, 3.19, and 3.93 mM). A volume of 250 μL of diluted h-CTZ was injected for each dose concentration at a rate of 1 ml/min. For the Injection Rate experiment, we injected 250 μL of h-CTZ at a concentration of 3.93 mM and rates of 0.1, 0.2, 0.3, or 1 ml/min.

### 2.4. Bioluminescence and fluorescence imaging

Imaging was done through the thinned skull using an EMCCD camera (Falcon III; Raptor Photonics) fitted with a 2X objective from Thorlabs while the mouse was awake, head-fixed, and running on a 3D-printed wheel in a light-proofed chamber that was built in-house. The timeline of events is illustrated in Fig. 1C. Prior to bioluminescence imaging, the mouse was put under light anesthesia to insert a tail vein catheter to deliver h-CTZ (Instech, Manufacturer Part Number: C10SS-MTV1417P). The lightly anesthetized plane was induced by placing the animal in an isoflurane chamber (~1.5 to 2 % induction) and was maintained with isoflurane through a nose cone (~1 %) for the duration of catheter placement. After placing the catheter in the tail vein, the animal was allowed to recover before the start of the imaging experiment (Fig. 1D). We injected the fluorescein dye (Ak-Fluor 10 %) to confirm that the tail vein catheter was placed correctly. After observing fluorescence emitted by Ak-Fluor, the catheter was taped firmly in place, and anesthesia was removed. Bioluminescence imaging was performed approximately 20 min after the brief anesthesia protocol to let the animal recover and be fully awake. Unless noted, bioluminescence was imaged with 60 s exposure time, and with the EM-gain of the EMCCD camera set at 3000. Thus, based on our imaging acquisition rate, time = ‘0′ represents the aggregated bioluminescence activity between 0 and 60 s post h-CTZ injection. In follow-up studies, we imaged bioluminescence activity with a faster exposure time < 10 s to study the temporal dynamics of the bioluminescence response at a finer detail. We also imaged fluorescence in response to injections of Ak-Fluor 10 % via the tail vein to estimate the time that it takes a chemical substance to reach the brain (exposure time = 0.5 Hz). The camera was connected to a chiller (Solid State Cooling Systems, UC160) with thermoelectric cooling temperature set to −70 °C to minimize noise emitted from heat mechanisms. Micromanager was used to control the EMCCD camera (Edelstein et al., 2010). We collected ten minutes of images (i.e., 10 frames) before injecting the h-CTZ. On frame eleven, we injected h-CTZ via the infusion pump, and imaging was continued for fifty additional minutes (i.e., 50 extra frames). Fig. 1D shows a representative example of the averaged bioluminescence map across one imaging session.

For the Dose Response experiment, we imaged bioluminescence in each mouse in response to different concentrations of h-CTZ (0.74, 0.98, 1.72, 2.46, 3.19, and 3.93 mM, at a rate of 1 ml/min). The order of h-CTZ dosage was randomized across each animal, with multiple h-CTZ injections performed on each day (e.g., Day 1 = 0.74, 2.46, and 3.93 mM; Day 2 = 3.19, 3.93, and 0.98; and Day 3 = 3.93, and 1.72). Thus, we derived a dose-response curve for each animal. Note that the highest h-CTZ concentration was delivered on each experimental day (i.e., repeated three times) in case we needed to normalize the bioluminescence response across days. The data associated with the highest h-CTZ condition was aggregated by taking the median across the three session values. Post-hoc analyses show that the normalization procedure was not necessary. The remaining h-CTZ dosages (i.e., < 3.93 mM) were injected only once per mouse. Daily experiments were performed approximately a week apart from each other. The highest concentration of h-CTZ was injected in each of the daily experiments. For the Injection Rate experiment, mice underwent only one experimental imaging day, with four different imaging sessions of varying injection rates (0.1, 0.2, 0.3, or 1 ml/min) using the same concentration of h-CTZ (3.93 mM) for each injection rate condition.

### 2.5. Histology

Mice were euthanized with isoflurane and perfused transcardially with 4 % paraformaldehyde (PFA). The brain was removed and stored in PFA at 4 °C for 36 h after perfusion. The brain was cryoprotected in 30 % sucrose for another 36 h prior to tissue slicing. Brains were sectioned in 40 μm slices on a cryostat (Leica CM1850), and mounted on glass slides in mounting medium (Vectashield Vibrance with DAPI; Vector Laboratories, H-1800) and imaged using a widefield Zeiss Microscope.

### 2.6. Imaging analyses

Images were taken at 16-bit resolution, and analyzed using custom-based scripts in MATLAB. For each mouse, a bioluminescence region of interest (ROI) was computed by drawing an area that contained the most amount of bioluminescence (Gomez-Ramirez et al., 2020). Note that the same ROI was used for all experimental conditions. Baseline activity was derived by averaging the signal in the ROI across the first ten frames of each experimental condition (i.e., 600 s). Baseline activity was then subtracted from each frame collected during the experiment.

Peak bioluminescence was calculated by averaging the image frame with maximum activity together with its two nearest neighboring frames. We performed randomization tests to statistically determine the onset and offset of bioluminescence activity in each mouse. Surrogate distributions (*N* = 5000) for estimating the onset of bioluminescence were generated by averaging across one hundred randomly sampled pixels from the first ten frames in the experiment (i.e., prior to h-CTZ injection). The initial frame that showed bioluminescence was estimated by determining the first two consecutive frames from the observed data that showed higher bioluminescence relative to the surrogate baseline distribution (probability value < 0.05). To estimate the offset of bioluminescence, we built surrogate distributions using image pixels that were randomly sampled from the last eight frames in the imaging session. Offset bioluminescence was estimated as the first frame of the observed data (after h-CTZ injection) that failed to show greater bioluminescence relative to the surrogate distribution built from the last eight frames in the session. Time of decay of the bioluminescence response was calculated as the time difference between the peak bioluminescence response and the bioluminescence offset. Area under the curve (AUC, i.e., total bioluminescence) was calculated by averaging activity across the start and end frame of the observed data. For the highest concentration in the Dose Response experiment, the response and time dynamics of bioluminescence was estimated by averaging across the values in each of the three sessions. Statistical testing at the group level was performed using Friedman repeated measures tests.

A Friedman test was used, instead of a parametric ANOVA, because the bioluminescence responses were not normally distributed, and our sample size was relatively small (*N* = 8). Effect sizes were estimated using Kendall’s W statistic described by Eq. (1):

(1) $$W=\frac{X^{2}}{n\times \left(k-1\right)}$$

Where *X^2^* = the Friedman test statistic value, *k* = the number of groups, and *n* = the total number of observations. Follow-up tests were done using Mann–Whitney U tests or curve fitting using a linear, quadratic, or exponential function. The model that provided 25 % higher adjusted-R^2^ values, relative to the adjusted-R^2^ of the linear function, was determined to be the model that best explains the data. However, if none of the models provided an adjusted-R^2^ greater than 0.1, then we deemed that none of the models were reliable predictors of the data.

## 3. Results

### 3.1. The bioluminescence response is proportional to luciferin dosage

**Fig. 2.**
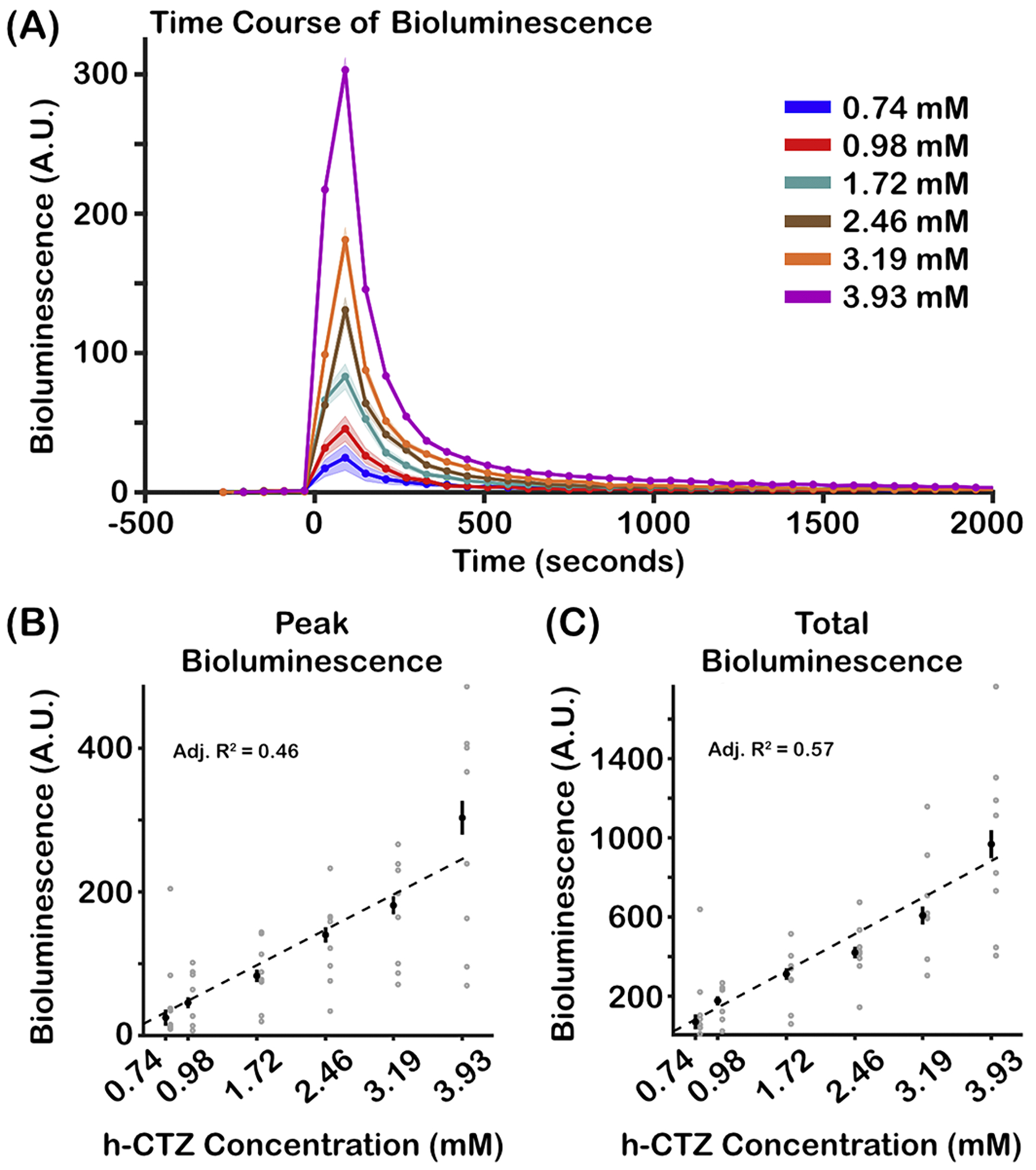
Bioluminescence response as a function of h-CTZ concentration. **(A)** Time course of Bioluminescence across h-CTZ concentration. Time point zero indicates the onset of the h-CTZ injection. Because of the course sampling of bioluminescence (i.e., 60 s exposure times), each dot point is plotted in between consecutive time frames. For example, the first point after the zero time point in the 3.93 mM condition corresponds to 30 s (i.e., the middle point between 0 s and 60 s). **(B)** Average bioluminescence around the peak (± 1 frame) for each mouse across h-CTZ concentration. **(C)** Total bioluminescence response, estimated as the area under the curve between the start and end points of bioluminescent activity. The black dots indicate the median bioluminescence values across animals, whereas the gray dots indicate each individual mouse’s response. Note that the gray dots have been shifted by 0.05 (around the x-axis) to provide better visibility of all the individual data points. The dash lines represent the best curve fit to the data. Error bars indicate standard error of the mean (SEM). Number of mice = 8.

Bioluminescence activity systematically increases as a function of h-CTZ dosage concentration. Fig. 2A shows the average bioluminescence time course for each h-CTZ concentration injected at 1 ml/min. The traces are time-locked to the onset of the injection. A Friedman test revealed significant differences in the bioluminescence peak response across h-CTZ conditions (X^2^(5) = 33.71, *p* = *2.72* × *10^−6^*; *W* = 0.84; Fig. 2B), with bioluminescence having a linear relationship to the dosage of the luciferin (adjusted-R^2^ = 0.46; Fig. 2B *dashed lines*). Note that a W greater than 0.5 is considered a large effect size. We also observed significant differences between h-CTZ dosage and area under the curve (AUC, defined as the mean activity between the onset and offset bioluminescence response; X^2^(5) = 33.43, *p* = *3.91* × *10^−6^*; *W* = 0.84; Fig. 2C). Similar to the bioluminescence peak response, we observed a linear relationship between the AUC and h-CTZ (adjusted-R^2^ = 0.57; Fig. 2C *dashed line*). Taken together, these data highlight the consistency and predictability of the bioluminescence response and, by proxy, BL-OG effects to a particular h-CTZ concentration.

### 3.2. Temporal dynamics of bioluminescence are consistent across luciferin dosage

**Fig. 3.**
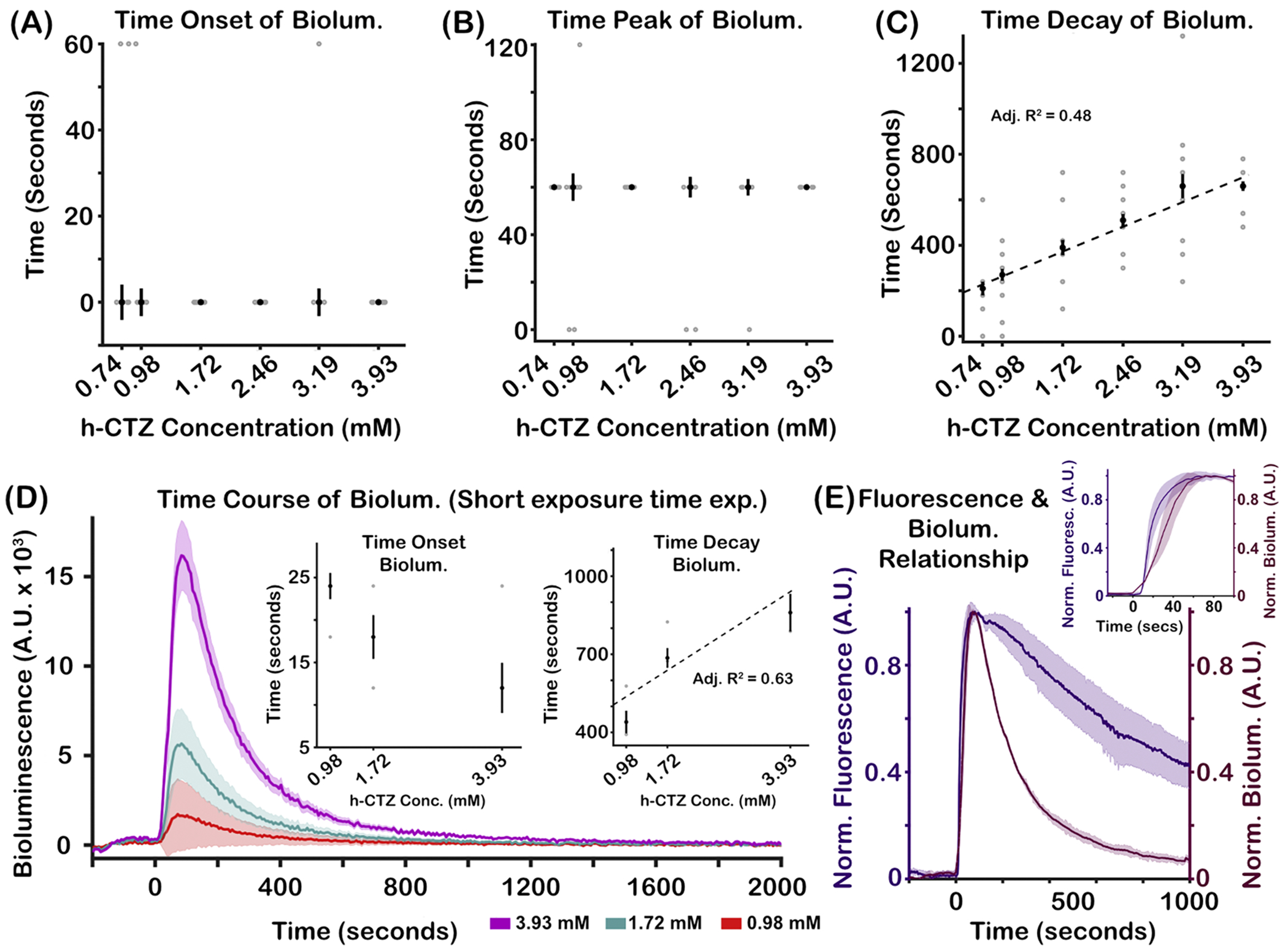
Temporal dynamics of bioluminescence response as a function of h-CTZ concentration. **(A)** Onset time of Bioluminescence across h-CTZ concentration. **(B)** Time of the bioluminescence peak response across h-CTZ concentration. The gray dots in (A) and (B) have been shifted by 0.05 (around the x-axis) to provide better visibility of all the individual data points. **(C)** Time of decay of the bioluminescence response (i.e., tau) across h-CTZ concentration. **(D)** Time course of Bioluminescence across three h-CTZ concentration levels using a short exposure time for imaging (6 s). Time point zero indicates the onset of the h-CTZ injection. The inset graphs show the time of bioluminescence onset (*left graph*) and time of bioluminescence decay (*right panel*) across injection rates. **(E)** Time course of fluorescence (*left axis*, from IV injection of Fluorescein) and bioluminescence (*right axis*) collected using exposure times of 0.5 and 10 s, respectively. The data are aligned to the onset time of the fluorescein and h-CTZ injections. The inset shows a zoomed-in version of the fluorescence and bioluminescence activity between −25 and 100 ms post injection. For all panels, the black dots indicate median times of bioluminescence across animals, whereas the gray dots indicate time values for each individual mouse. The dash lines represent the best curve fit to the data. Note that for Figs. 3A and 3B, the curve fitting analyses did not provide a reliable fit. Error bars indicate SEM. Figs. 3A, 3B, and 3C number of mice = 8. Fig. 3D number of mice = 3. Fig. 3E number of mice = 7 (2 fluorescein experiments, and 5 bioluminescence experiments).

Systemic h-CTZ injection generates bioluminescent activity within the first 60 s for all dosage concentration. Fig. 3A shows the median onset time of bioluminescence for each h-CTZ concentration. A Friedman test did not reveal differences in bioluminescence onset time across h-CTZ conditions (X^2^(5) = 0.56, *p = 0.98*). A Friedman test also showed no significant differences in peak activation timing across all h-CTZ dosages (X^2^(5) = 1.32, *p = 0.93*; Fig. 3B), with the peak activity occurring between 60 and 120 s following h-CTZ injection. However, we found that the decay time of the bioluminescence response systematically increases as a function of dosage concentration (Fig. 3C). A Friedman test revealed significant differences in time decay between h-CTZ dosage (X^2^(5) = 33.21, *p* = *3.42* × *10^−6^*; *W* = 0.83), with bioluminescence having a linear relationship to h-CTZ dosage (adjusted-R^2^ = 0.48; Fig. 3C *dashed lined*). Bioluminescence was imaged using a 60 s exposure time to obtain a strong and reliable measure of the signal (see e.g., Gomez-Ramirez et al. 2020). However, this image acquisition rate provides a coarse estimate of the time onset properties of bioluminescence. As such, we performed additional imaging experiments where bioluminescence was sampled at a faster rate (i.e., a short exposure time of 6 s) to have a better estimate of the temporal dynamics of the bioluminescence response across h-CTZ dosage conditions. We increased the EM gain of the camera to its maximum to capture images with larger SNRs. These additional experiments were conducted on a separate cohort of mice (*N* = 3) who expressed the LMO7 molecule. Imaging was done via a thinned skull, and injecting a smaller set of the h-CTZ dosage conditions (0.98, 1.72, and 3.93 mM). Fig. 3D shows the time course of the bioluminescence in response to the three h-CTZ dosage conditions imaged with a short exposure time, with higher h-CTZ dosage generating greater bioluminescence activity. The *left inset* in Fig. 3D shows the onset time of bioluminescence for the three h-CTZ conditions imaged with a short exposure time. Although the largest condition seemed to have the shortest onset time, a Friedman test failed to show a significant difference in bioluminescence onset time between the dosage conditions (X^2^(2) = 2.17, *p = 0.34*). Although we imaged activity using the camera’s highest EM gain, the delayed onset time for the lower dosage conditions may be caused by lower SNR due to the faster sampling rate of imaging. In support, we observed that the peak onset time of bioluminescence was similar across the three dosage conditions (Peak Time = 66, 54, and 72 s for the 0.98, 1.72, and 3.93 mM dosages, respectively; X^2^(2) = 1.5, *p = 0.47*). We also observed that the time of decay increased as a function of h-CTZ dosage (X^2^(2) = 6.26, *p* = *0.04*; adjusted-R^2^ = 0.63; *right inset* Fig. 3D). These findings from the short exposure time experiments are highly consistent with those results from experiments using a longer exposure time. Taken together, these data show that although the onset and peak responses of bioluminescence are consistent across dosage, the duration of the bioluminescence response systematically increases as a function of the luciferin concentration.

We performed an additional control experiment where we injected fluorescein via the tail vein in 3 mice to determine the time it takes for the injected substrate to reach the brain. Fig. 3E shows the time course of the normalized fluorescence response (left axis, purple trace), and normalized bioluminescence response to the highest h-CTZ dosage (3.93 nM; right axis, burgundy color). Three mice were imaged with an exposure time of 6 s, and two mice were imaged with a 10 s exposure time. The median onset time of bioluminescence is a bit delayed relative to the fluorescence onset from the fluorescein injection (18 vs. 9.6 s; see Fig. 3E *inset*), but bioluminescence still occurred relatively quickly after the h-CTZ injection. Taken together, these data indicate that systemic injections of luciferin generate rapid bioluminescence responses in the brain.

### 3.3. Bioluminescence dynamics are largely stable across luciferin injections with different rates

**Fig. 4.**
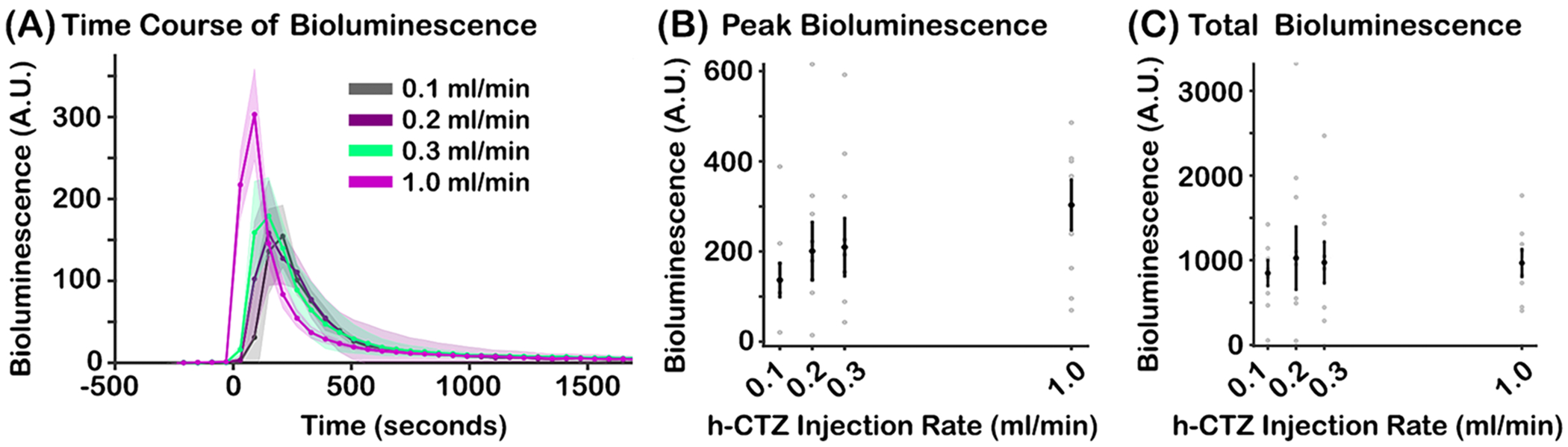
Bioluminescence response as a function of h-CTZ injection rate. **(A)** Time course of bioluminescence across rate of injection of h-CTZ (at 3.93 mM concentration). Time point zero indicates the onset of the h-CTZ injection. Similar to Fig. 2A, each dot is plotted between consecutive time frames. The first point after the zero time point in the 1.0 ml/min condition corresponds to 30 s (i.e., the middle point between 0 s and 60 s). **(B)** Average bioluminescence response around the peak (± 1 frame) for each mouse across h-CTZ injection rate. **(C)** Total bioluminescence response, estimated as the AUC between the start and end points of bioluminescent activity. The black dots indicate the median bioluminescence values across animals, whereas the gray dots indicate each individual mouse’s response. The dash lines represent the best curve fit to the data. Note that the peak and AUC of the bioluminescence response were constant across rate conditions, thus the fits were not reliable. Error bars indicate SEM. Number of mice = 6 for injection rate conditions 0.1 – 0.3 ml/min. Number of mice = 8 for injection rate condition 1.0 ml/min.

Bioluminescence activity is similar regardless of the injection rate of h-CTZ of high concentration (i.e., 3.93 mM). Fig. 4A shows the average bioluminescence time course for each h-CTZ injection rate. The traces are time-locked to the onset of the injection. A Friedman test did not reveal significant differences in peak responses (X^2^(3) = 3.19, *p* = *0.36*; Fig. 4B) or AUC activity (X^2^(3) = 2.36, *p* = *0.50*; Fig. 4C) across h-CTZ injection rate conditions. Thus, the strength of BL-OG effects appears to be insensitive to the injection rate of the luciferin.

### 3.4. Temporal dynamics of bioluminescence vary by rate of luciferin injection

**Fig. 5.**
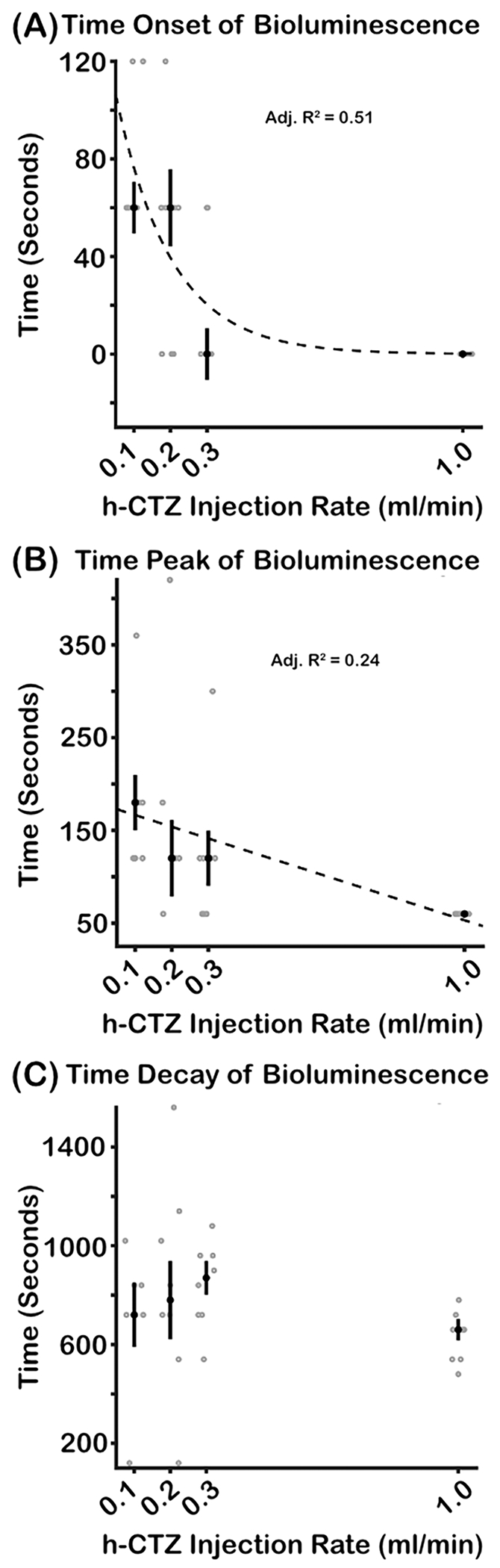
Temporal dynamics of bioluminescence response as a function of h-CTZ injection rate. **(A)** Onset time of the bioluminescence response across rate of injection of h-CTZ (at 3.93 mM concentration). **(B)** Time of the bioluminescence peak response across h-CTZ injection rate. **(C)** Time of decay of the bioluminescence response (i.e., tau) across h-CTZ injection rate. The black dots indicate median times of bioluminescence across animals, whereas the gray dots indicate time values for each individual mouse. The dash lines represent the best curve fit to the data. Note that the time of decay of the bioluminescence response was constant across rate conditions, thus the fit was not reliable. Error bars indicate SEM. Number of mice = 6 for injection rate conditions 0.1 – 0.3 ml/min. Number of mice = 8 for injection rate condition 1.0 ml/min.

Injection rate of h-CTZ systematically modulates the onset of bioluminescent activity. Fig. 5A shows the median onset time of bioluminescence for each injection rate condition. A Friedman revealed significant differences in bioluminescence onset time across injection rate (X^2^(3) = 12.97, *p* = *0.043*; *W* = 0.54), with the onset time exponentially decreasing as a function of injection rate (adjusted-R^2^ = 0.51; Fig. 5A *dashed lines*). The data further show that the peak time of activation also decreases as a function of injection rate (X^2^(3) = 13.05, *p* = *0.0045; W* = 0.54; Fig. 5B), with the bioluminescence peak time linearly decreasing with injection rate speed (adjusted-R^2^ = 0.24; Fig. 5B *dashed lines*). However, the data did not show an effect of injection rate on the time decay of bioluminescence (X^2^(3) = 3.79, *p* = *0.285,* Fig. 5C). These data reveal that the early dynamics of the bioluminescence response are systematically modulated by the rate of injection of the luciferin.

### 3.5. Bioluminescence activity is specifically generated by the LMO7 molecule

**Fig. 6.**
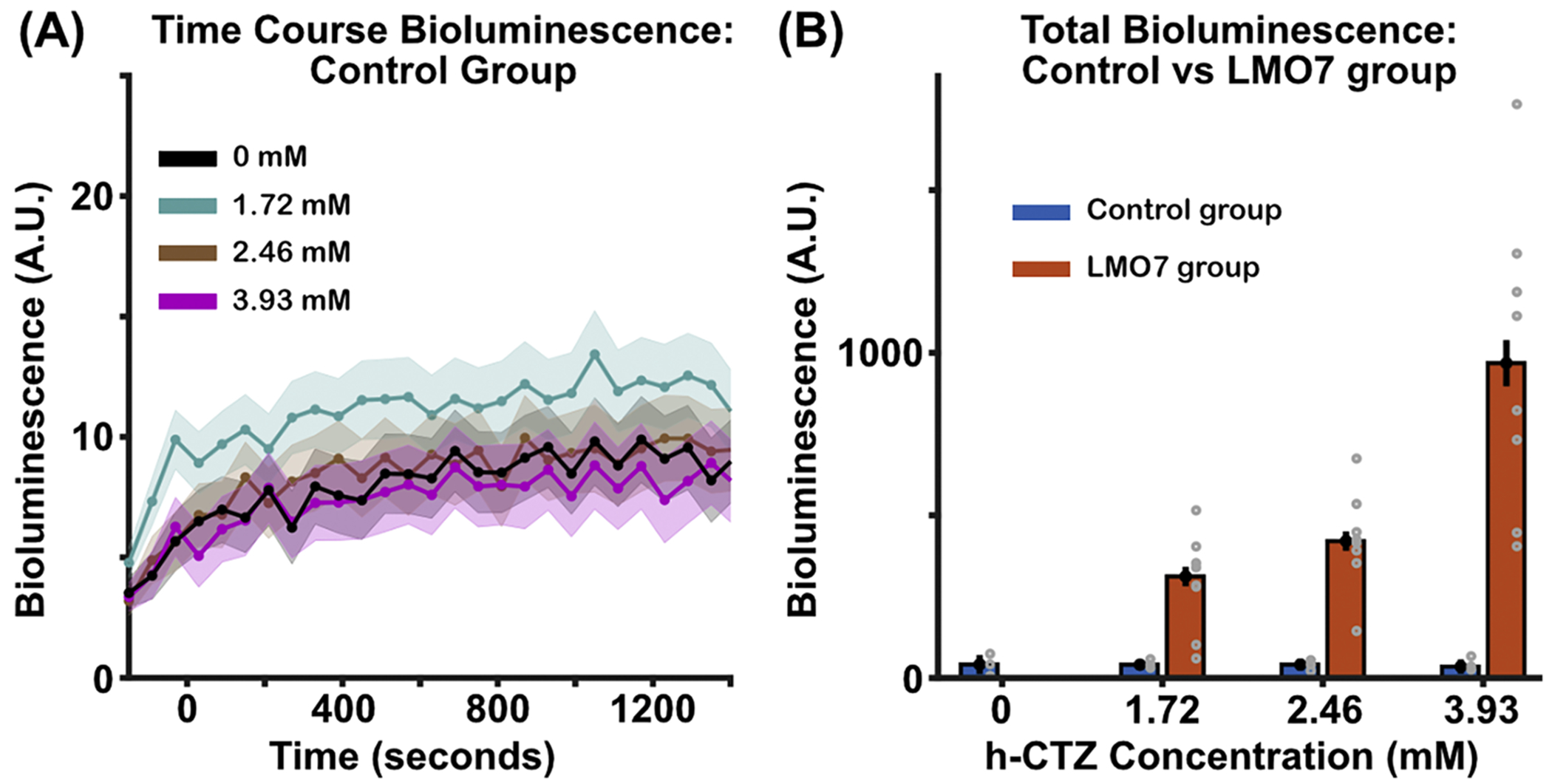
Bioluminescence control experiments **(A)** Time course of bioluminescence across three h-CTZ dosage conditions (0, 1.72, 2.46, and 3.93 mM) in animals not expressing a bioluminescent molecule. Time point zero indicates the onset of the h-CTZ injection. In the 0 mM condition, animals were not injected with h-CTZ to determine ‘photonic noise’ from the EMCCD camera. **(B)** Area under the curve of the bioluminescence activity in the Control (*blue bars*) and LMO7 (*orange bars*) groups. The black dots indicate the median bioluminescence values across animals, whereas the gray dots indicate each individual mouse’s response. Note that the gray dots have been shifted by 0.05 (around the x-axis) to provide better visibility of all the individual data points. Error bars indicate SEM. Number of mice in control and LMO7 groups are 3 and 8, respectively.

We performed an additional control experiment to determine how much of the bioluminescence response is driven by ‘auto-bioluminescence’ of the h-CTZ itself (i.e., uncaging of photons not created by the luciferase/luciferin interaction). In these control experiments, we imaged activity in three mice that did not express LMO7. Three different concentrations of h-CTZ were injected via the tail vein (1.72, 2.46, and 3.93 mM). We also imaged activity without injecting h-CTZ (i.e., 0 mM dosage) to estimate the ‘photonic noise’ of the EMCCD camera. Fig. 6A shows very weak photonic activity for all h-CTZ dosage conditions. However, we observed similar values for the 0 mM dosage condition (X^2^(3) = 1, *p* = *0.8013*), indicating that this weak bioluminescence activity is driven by ‘photonic noise’ from the EMCCD camera. Fig. 6B shows that total bioluminescence (area under the curve) across h-CTZ dosages for the control group vs. the mouse cohort expressing the LMO7 molecule. These data show a ~15 fold increase in the bioluminescence response observed in the LMO7 vs. the control group (averaged across h-CTZ dosages). These data indicate that the bioluminescence activity observed in animals expressing the LMO7 molecule is not produced by ‘auto-bioluminescence’ from h-CTZ.

## 4. Discussion

We characterized dose response functions and temporal dynamics of bioluminescence in response to different h-CTZ injection parameters in mice expressing the BL-OG molecule LMO7, a fluorescent protein-luciferase fusion tethered to VChR1. Bioluminescence was imaged through a thinned-skull, while delivering injections of h-CTZ via the tail vein. The data show that the strength of bioluminescence, measured during the peak activity and AUC (i.e., the total bioluminescence across time), linearly increases as a function h-CTZ concentration (see Figs. 2B and 2C).

Importantly, the peak and AUC of the bioluminescence response is unaffected by the rate of injection of h-CTZ (see Fig. 4B and 4C). Future projects using single-unit electrophysiology or 2-photon fluorescence imaging will determine whether these concentration-dependent increases in BL-OG bioluminescence lead to more neurons being recruited and/or amplifications of individuals’ neural firing themselves.

The findings in this study build on our previous work that show robust and systematic bioluminescence activity in response to direct injection of the luciferin in the brain (Gomez-Ramirez et al., 2020). Here, we further demonstrate this robust and parametric relationship between the strength of the bioluminescence response and luciferin dosage using systemic injections of the luciferin. Our data also reveal that bioluminescence activity produced by the BL-OG molecule can be reliably imaged through a thinned skull of a mouse, highlighting BL-OG’s feasibility to track neuromodulation effects through minimally invasive procedures. In sum, our study provides strong evidence that IV injections of h-CTZ generate concentration-dependent BL-OG effects in the brain, and that these effects are independent of the rate at which the luciferin is delivered.

### 4.1. Temporal dynamics of the bioluminescence response

The onset time of bioluminescence in neocortex is rapid and invariant to h-CTZ concentration (see Fig. 3A). In particular, bioluminescence emerges within the first 60 s after h-CTZ injection in all concentration conditions. However, using shorter exposure times (i.e., higher sampling rates), we found that the onset time of bioluminescence is ~18 s after h-CTZ injection. This rapid onset time highlights the robust bioavailability of h-CTZ to the brain, and the fast-acting mechanisms of BL-OG to elicit neuromodulation effects. We also found that the onset peak response is unaffected by the concentration of h-CTZ. Further, as expected, the decay time of bioluminescence linearly increases as a function of h-CTZ concentration, likely due to the large amount of the substrate that is catalyzed by the luciferase.

As expected, the onset time of bioluminescence modulates as a function of injection rate. Specifically, we observed a nonlinear relationship (exponential) between bioluminescence onset and injection rate, with faster rates generating earlier onset times (see Fig. 5A). We note that, although bioluminescence onset times are fit well by an exponential function, the pattern may be driven (at least in part) by the sparse sampling in the imaging and injection rate conditions. As such, future experiments should sample bioluminescence activity much faster to determine whether the onset of bioluminescence activity in the brain scales linearly vs. non-linearly (e.g., exponentially) with the rate of injections of the luciferin. The data also show that the peak time of bioluminescence decreases as the rate of injection is increased (see Fig. 5B). However, the decay time of bioluminescence is unaffected by injection rate. Thus, our data indicate that the rate of injection of h-CTZ modulates the bioluminescence response by shifting the onset and peak activation time, without modifying the decay dynamics of the bioluminescence response. These effects of injection rate are notable because they generate bioluminescence activations (e.g., AUC responses) that are largely homogenous regardless of the injection rate (see e.g., Fig. 4C).

The onset time of BL-OG effects appear to be substantially faster than the neuromodulation onset times of other chemogenetic methods such as DREADDs (Deffains et al., 2021; Nagai et al., 2020). For instance, it was shown that deschloroclozapine (DCZ) administered IV at a rate of 0.2 ml/*sec* leads to neuromodulation effects that commence around 5 min after injection (Nagai et al., 2020). In contrast, using the same injection rate of h-CTZ, we observed bioluminescence occurring as early as 18 s after injection (see Fig. 3D). The difference in neuromodulation onset times between the two methods is unclear, but DREADDs operate on secondary messenger systems (i.e., G protein-coupled receptors; GPCRs) which have significantly slower kinetics as compared to ionotropic channels in which optogenetic elements operate on (Armbruster et al., 2007; Whissell et al., 2016). Our data also show that BL-OG effects can be short-lasting (~3 to ~12 min), providing repeated BL-OG-mediated neuromodulation effects within a single experimental day. In contrast, the duration of the neuromodulation effects of DREADDs are thought to last hours (Alexander et al., 2009; Whissell et al., 2016). Taken together, our data demonstrate that the duration of BL-OG effects can be regulated by the concentration amount, volume (see Gomez-Ramirez et al., 2020), or injection rate of the luciferin, indicating that the dynamics of neuromodulation effects of BL-OG can be systemically controlled by experimenters.

Our study provides a generalized framework of the experimental parameters that control the strength and dynamics of BL-OG effects generated by the LMO7 molecule in the brain. In particular, our data provide evidence that BL-OG effects driven by LMO7 can be fast, with the strength and duration of the neuromodulation effects modulated by the injection parameters of the luciferin. Our data further show that the BL-OG method is unique in providing online tracking of the neuromodulation effects via the bioluminescent activity, and this tracking can be achieved in lieu of chronically-implanted cranial windows. In sum, this study highlights the reliability of BL-OG as a major neuromodulation method and demonstrates its feasibility to monitor neuromodulation effects through minimally invasive approaches.

## Data Availability

All data reported in this paper are stored in Neurodata Without Borders (NWB) format, and will be shared by the corresponding author upon request. Any additional information required to reanalyze the data reported in this paper is available from the corresponding author upon request. It is expected that publications produced from a shared data agreement would result in co-authorships for all of the authors in this manuscript.
